# Malignant Peripheral Nerve Sheath Tumour of the Maxilla

**DOI:** 10.1155/2014/230849

**Published:** 2014-03-18

**Authors:** Puja Sahai, Bidhu Kalyan Mohanti, Devajit Nath, Suman Bhasker, Subhash Chander, Sameer Bakhshi, Chirom Amit Singh

**Affiliations:** ^1^Department of Radiation Oncology, Dr. B.R.A Institute Rotary Cancer Hospital, All India Institute of Medical Sciences, Ansari Nagar, New Delhi-110029, India; ^2^Department of Pathology, All India Institute of Medical Sciences, New Delhi, India; ^3^Department of Medical Oncology, Dr. B.R.A Institute Rotary Cancer Hospital, All India Institute of Medical Sciences, New Delhi, India; ^4^Department of Otorhinolaryngology—Head and Neck Surgery, All India Institute of Medical Sciences, New Delhi, India

## Abstract

A 38-year-old man was diagnosed with malignant peripheral nerve sheath tumour of the maxilla. He was treated with total maxillectomy. Histopathological examination of the resected specimen revealed a close resection margin. The tumour was of high grade with an MIB-1 labelling index of almost 60%. At six weeks following the surgery, he developed local tumour relapse. The patient succumbed to the disease at five months from the time of diagnosis. The present report underlines the locally aggressive nature of malignant peripheral nerve sheath tumour of the maxilla which necessitates an early therapeutic intervention. A complete resection with clear margins is the most important prognostic factor for malignant peripheral nerve sheath tumour in the head and neck region. Adjuvant radiotherapy may be considered to improve the local control. Future research may demarcate the role of targeted therapy for patients with malignant peripheral nerve sheath tumour.

## 1. Introduction

Malignant peripheral nerve sheath tumours (MPNSTs) encompass a cluster of tumours which arise from the peripheral nerves or display differentiation along various elements of the nerve sheath, namely, Schwann cells, perineural fibroblasts, or fibroblasts [1]. An origin of these tumours in the head and neck region is uncommon occurring in approximately 10% of all cases [2, 3]. Herein, a case of maxillary MPNST is discussed along with the literature review of head and neck MPNSTs.

## 2. Case Report

A 38-year-old man presented with an 8-month history of pain in the left cheek, bleeding from the left nostril, and nasal blockage. He had noticed a swelling over the left cheek and dorsum of the nose which progressed over a period of 6 months. He had also noticed an ulcer on the left upper alveolus which progressed over a period of 2 months. He had a 10-year history of tobacco consumption. A contrast enhanced computed tomography (CECT) of the paranasal sinuses (PNS) was performed which revealed a mass lesion in the left maxillary sinus. He had undergone a surgery for the removal of mass in the left maxillary antrum and nasal cavity by Caldwell Luc approach. Histopathological examination of the resected mass was reported as angiofibroma. After remaining asymptomatic for 5 months, the patient presented with the similar complaints as before for duration of one month. The patient was now evaluated in our multidisciplinary clinic by a team of head and neck surgeon, radiation oncologist, and a medical oncologist. Physical examination revealed swelling of 8 × 6 cm size over the left cheek involving the nasolabial fold. There was no abnormality seen on the overlying skin. No sensory deficit was seen in the region of the trigeminal nerve distribution. Oral cavity examination revealed a polypoidal friable mass lesion arising from the left upper alveolus. A similar mass lesion was seen filling the left nasal cavity. A bulge was seen in the region of the hard palate with prominent overlying vessels. The patient did not have any signs of neurofibromatosis.

Magnetic resonance imaging (MRI) of the PNS and neck was performed which revealed an ill-defined soft-tissue mass lesion in the left maxillary sinus. The lesion showed isointense and hyperintense appearance on T1- and T2-weighted images, respectively. A few hypointense areas were noted in the mass lesion suggestive of spicules of destroyed bone. There was no haemorrhage or necrosis seen within the lesion. The mass lesion showed contiguous extension to the left nasal cavity with destruction of the middle and inferior turbinate. Cranially, the lesion was seen reaching till the left ethmoid sinus with destruction of air cells. Caudally, the lesion was extending to the hard palate with destruction of the floor of the maxillary antrum. The anterior wall of the maxilla was destroyed with extension of the mass lesion into subcutaneous plane of the premaxillary area. The posterior wall of the maxillary antrum was destroyed at places with extension of the mass lesion into retromaxillary fat pad. Infiltration of the left maxillary alveolar arch by the lesion was seen. The mass lesion demonstrated restricted diffusion on the diffusion-weighted images. There was no intraorbital or intracranial extension. No significant cervical lymphadenopathy was seen. A CECT of the PNS was also performed which showed a mass lesion measuring 7.6 × 4.7 cm in the left maxillary sinus extending to the ethmoidal air cells, nasal cavity, pterygomaxillary fissure, and pterygopalatine fossa. Irregularity of the left pterygoid plates was seen. Destruction of the left palatine bone and maxillary alveolar process in the region of premolars and molars was seen with eroded buccal and palatal cortices. Chest radiograph demonstrated normal lung fields.

**Figure 1:**
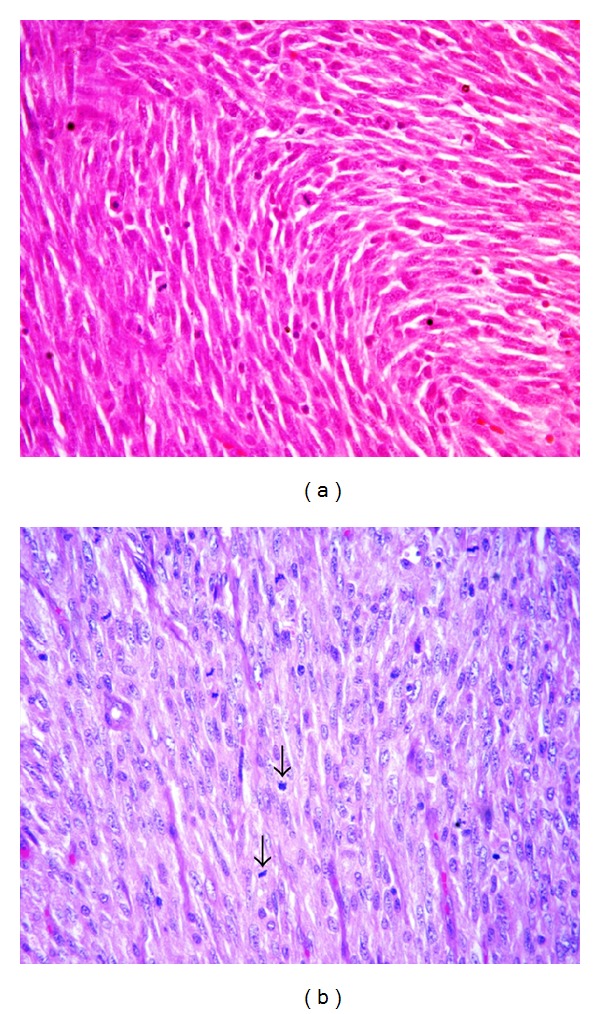
Haematoxylin and eosin stained sections show plump to spindle-shaped tumour cells with eosinophilic cytoplasm and focal hyperchromatic wavy nuclei arranged in whorls ((a), ×40); arrows denote mitotic activity ((b), ×40).

**Figure 2:**
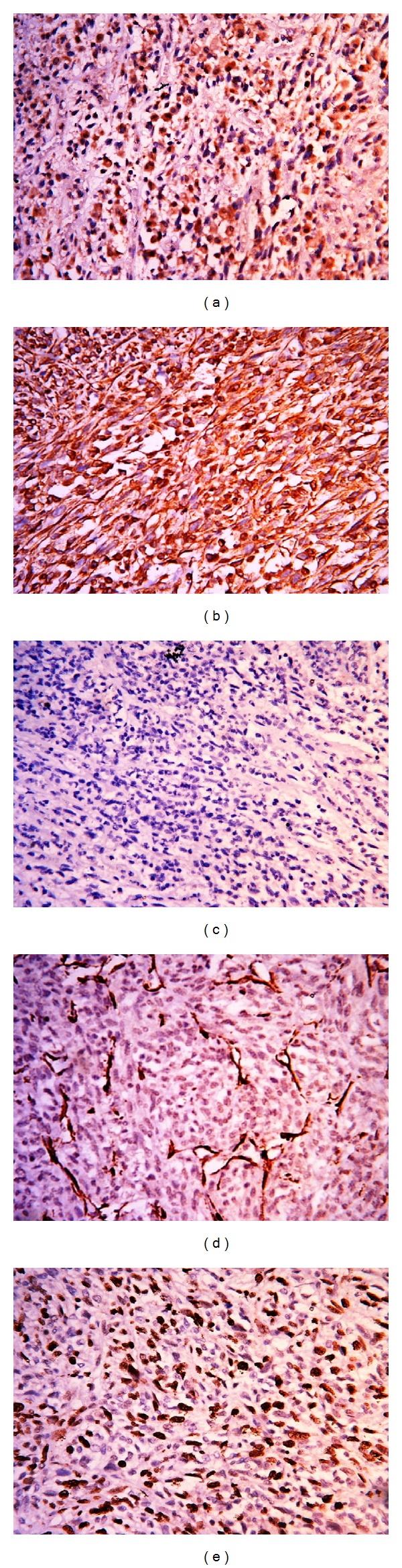
The tumour cells show nuclear immunopositivity for S100 ((a), ×40), diffuse positivity for vimentin ((b), ×40), immunonegativity for CD99 ((c), ×40), smooth muscle actin ((d), ×40), and high MIB-1 labeling index ((e), ×40).

A biopsy of the mass lesion was performed. Histopathological examination of the biopsied specimen revealed features of malignant mesenchymal tumor. The tumour cells demonstrated immunopositivity for vimentin and immunonegativity for cytokeratin, epithelial membrane antigen, desmin, myogenin, and smooth muscle actin (SMA). Subsequently, the patient underwent a total left maxillectomy. Peroperative findings revealed a proliferative growth in the left maxilla and nasal cavity reaching up to nasopharynx and infratemporal fossa. Gross examination of the resected specimen revealed a tumour measuring 8 × 3 × 5 cm. There were also separately lying pieces of tumour tissue collectively measuring 4 × 3 × 2 cm. The tumour was lying close to the right lateral mucosal margin and 0.5 cm from the nearest bony resected margin. On microscopic examination, the right lateral mucosal resection margin was 0.3 cm. The remaining mucosal and bony resected margins were free of tumour. Spindle-shaped atypical cells with a high mitotic activity (15/10 hpf) were seen on histopathological examination (Figure 1). No necrosis was seen. The tumour cells demonstrated diffuse positivity for vimentin and patchy nuclear positivity for S100 (Figure 2). The cells showed immunonegativity for MIC-2, BCL-2, CD34, cytokeratin, CD99, and SMA (Figure 2). The MIB-1 labelling index was almost 60% (Figure 2). Based on the aforementioned findings, a pathological diagnosis of high grade MPNST was made.

**Figure 3:**
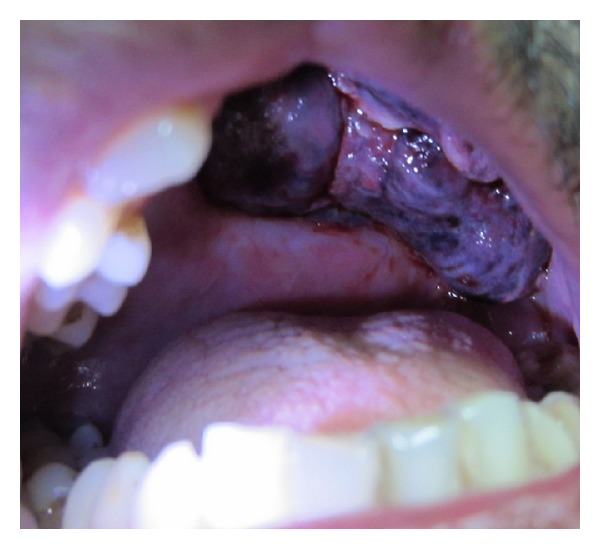
Clinical photograph shows a proliferative mass lesion arising at the maxillectomy site.

**Figure 4:**
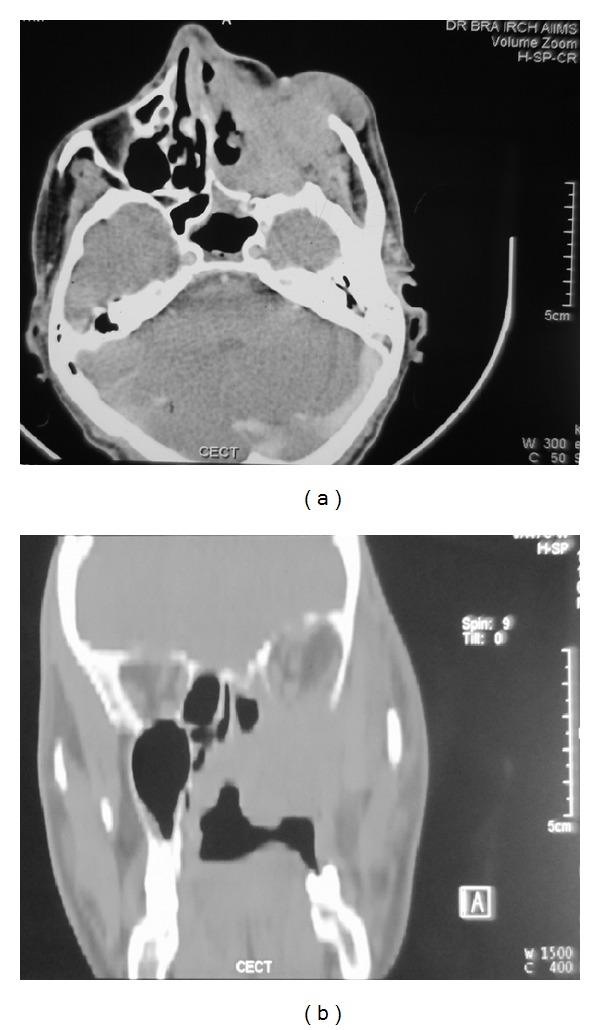
Axial (a) and coronal (b) images of contrast enhanced CT of the paranasal sinuses show an ill-defined poorly enhancing lesion at the operated (maxillectomy) site extending into the left orbit.

As part of metastatic work-up, ^99m^Tc-methylene diphosphonate planar (MDP) bone scintigraphy with single photon emission computed tomography-computed tomography (SPECT-CT) was performed. There were no skeletal metastases evident on the scan. Adjuvant radiotherapy was planned for the patient. However, at six weeks subsequent to the surgery, he presented with a mass lesion in the oral cavity at the maxillectomy site (Figure 3). His performance status was ECOG 2. A biopsy was performed from the mass lesion, which revealed a malignant tumour of similar morphology as the previous one. In view of the local tumour relapse, adjuvant radiotherapy could not be delivered. A CECT of the PNS was performed which demonstrated an ill-defined poorly enhancing lesion measuring 7 × 5 cm at the operated site (Figure 4). The lesion was seen extending to the left ethmoid air cells and orbit with destruction of the medial wall of the latter. The lesion was seen infiltrating into the left infratemporal fossa and masticator space. Posteromedially, the mass lesion was seen protruding into nasopharynx and oropharynx. No cervical lymphadenopathy was seen. A CT of the chest was performed in which no lung metastases were detected. Salvage chemotherapy was planned for the patient. He received one cycle of chemotherapy with the following regimen: ifosfamide 1.8 mg/m^2^ IV with mesna D1–D5 and doxorubicin 25 mg/m^2^ IV D1–D3. However, no regression of the mass lesion was seen. The patient succumbed to the disease at five months from the time of diagnosis.

## 3. Discussion

The MPNSTs are classified as low or high grade based on the cellular differentiation, mitotic count, and expression of immunohistochemistry markers. Low grade MPNSTs display diffuse or focal reactivity for S100, CD57, CD34, p16, and p27 while negative reactivity for p53, MIB-1, and topoisomerase II-alpha (TopoIIalpha) [4, 5]. The cellular staining of MIB-1 proliferation marker in low grade MPNSTs ranges from 1% to 5% [4]. Most high grade MPNSTs display decreased or negative reactivity to S100, CD57, CD34, p16, and p27 while an increased reactivity to p53, MIB-1, and TopoIIalpha [5].

Minovi et al. [6] reported the outcome of 10 patients with head and neck MPNST seen over a span of 20 years. Two patients had primary site involvement in paranasal sinuses. One patient was treated with preoperative chemotherapy with no evidence of tumour regression. Recurrence was seen in one patient while receiving postoperative radiotherapy. The 5-year survival rate was estimated at 20%. The published case series and reports pertaining to the head and neck MPNSTs have been summarized by Minovi et al. in their article. The statistics confined to the head and neck MPNSTs showed 5-year survival rates ranging from 15% to 47% [1, 7–10]. The published reports indicate that complete surgical removal of the MPNST is the mainstay of treatment and the most important prognostic factor [6, 9, 10].

Bagan et al. [11] described a case of MPNST arising from the maxilla which was not associated with neurofibromatosis type 1 in a 12-year-old male. He was treated with hemimaxillectomy and adjuvant radiotherapy. The tumour was resected with negative resection margins. The patient developed local tumour relapse and died after 10 months of the primary surgery. Maheshwari et al. [12] reported a case of sinonasal malignant schwannoma in a 60-year-old man. He was treated with medial maxillectomy and ethmoidectomy with complete removal of the tumour followed by adjuvant radiotherapy to a dose of 60 Gy in 30 fractions over 6 weeks. The patient remained disease-free at the follow-up period of one year. A better survival has been reported in patients with MPNST treated with postoperative radiotherapy [13].

The survival meta-analyses by Kolberg et al. [14] reported the outcome in 179 patients with MPNST from three European sarcoma centers. The 5-year disease-specific survival, overall survival, and disease-free survival rates were 46%, 44%, and 37%, respectively. There was no statistically significant difference in the survival rates between patients with and without neurofibromatosis type 1. The clinical parameters, namely, tumour grade and size, surgical remission status, and metastatic disease at the time of initial diagnosis, were significantly associated with survival. Patients selected for chemotherapy seemed to have a worse prognosis. No impact on survival was seen with radiotherapy. Distant metastases have been reported in patients with MPNST resulting in an inferior survival [1, 15]. The best response rates have been documented with the ifosfamide and doxorubicin combination chemotherapy in patients with MPNST [16]. Recent preclinical studies suggest inhibition of phosphatidylinositol 3-kinase (PI3 K)/AKT and mTOR pathway as a potential therapeutic approach for patients harbouring MPNST [17, 18].

The present report underlines the locally aggressive nature of MPNST of the maxilla which necessitates an early therapeutic intervention. A complete resection with clear margins is the most important prognostic factor for MPNST in the head and neck region. Despite the conflicting reports, adjuvant radiotherapy may be considered to improve the local control. Future research may demarcate the role of targeted therapy for patients with MPNST.
